# Perception of Generic Drugs Among Pharmacists in Poland: The Role of Sociodemographic Factors in Shaping Professional Attitudes and Practices

**DOI:** 10.3390/healthcare13202629

**Published:** 2025-10-20

**Authors:** Marcin Lewandowski, Urszula Religioni, Dariusz Świetlik, Adam Kobayashi, Marcin Czech, Piotr Wierzbiński, Daniel Śliż, Waldemar Wierzba, Katarzyna Plagens-Rotman, Piotr Merks

**Affiliations:** 1 Department of Pharmacology and Clinical Pharmacology, Faculty of Medicine, Collegium Medicum, Cardinal Stefan Wyszyński University, 01-938 Warsaw, Poland; 2 School of Public Health, Centre of Postgraduate Medical Education, 00-416 Warsaw, Poland; 3 Division of Biostatistics and Neural Networks, Medical University of Gdańsk, 80-210 Gdańsk, Poland; 4 Department of Pharmacoeconomics, Institute of Mother and Child, 01-211 Warsaw, Poland; 5 Polish Society of Holistic Medicine, 00-388 Warsaw, Poland; 6 3rd Department of Internal Medicine and Cardiology, Medical University of Warsaw, 02-091 Warsaw, Poland; 7 Department of Digital Medicine, Implementation and Innovation, National Medical Institute of the MSWiA, 02-507 Warsaw, Poland; 8 Center for Pediatric, Adolescent Gynecology and Sexology, Division of Gynecology, Department of Gynecology, Poznan University of Medical Sciences, 61-701 Poznan, Poland

**Keywords:** generic medicines, pharmacists’ attitudes, sociodemographic determinants, bioequivalence, health policy, patient-reported outcomes

## Abstract

Background: Pharmacists’ perceptions and practices shape the real-world uptake of generic medicines. From a health-economics perspective, wider generic substitution reduces patient out-of-pocket spending and creates headroom in payer budgets for high-value interventions. We assessed attitudes toward the efficacy, safety, and use of generics and examined sociodemographic correlates among Polish pharmacists. Methods: Analytical cross-sectional survey of licensed pharmacists in Poland was used (June–August 2025). The questionnaire covered reasons for recommending generics in long-term and single-use therapy; doubts about efficacy; views on bioequivalence testing; patient-reported experiences; and Likert-scale opinions on innovation, safety, efficacy, access, and payer savings. Associations were tested with χ^2^ and Mann–Whitney U (α = 0.05). Results: Of 342 respondents (67.5% women; 74.9% community pharmacists), cost was the leading reason to recommend generics in long-term therapy (91.0%), followed by efficacy (53.0%) and safety (51.5%); for single-use prescriptions, cost remained central (76.2%), with lower emphasis on efficacy (47.5%) and safety (45.0%). Pharmacists who never recommend generics were older and more experienced (*p* = 0.006; *p* = 0.012). Doubts about generic efficacy were reported by 36.2% overall and more often among women, hospital pharmacists, and those with a specialization; 53.5% of those with doubts would advise switching even to a costlier option. Nearly half supported conducting bioequivalence studies between generics (49.6%). Positive perceptions predominated: 82.9% agreed generics are as effective and 84.6% as safe as originators. Most endorsed system benefits, including payer savings enabling list expansion (73.6%) and improved patient access (92.5%); agreement on access was higher among community pharmacists (*p* = 0.004). Conclusions: Polish pharmacists largely view generics as clinically equivalent and system-enhancing, with cost the dominant driver of recommendation. Targeted education—especially for hospital settings and specialized pharmacists—and attention to patient-reported experiences may further strengthen confidence and appropriate use of generics.

## 1. Introduction

The increasing demand for cost-effective pharmacological therapies in contemporary health systems has intensified the global discussion on how best to reconcile economic efficiency with clinical quality. As the costs of originator (brand-name) drugs continue to rise, both policymakers and healthcare professionals increasingly emphasize the importance of expanding the use of generic medicines. Generics, defined as pharmaceutical products containing the same active substances in the same dosage and pharmaceutical form as their branded counterparts, and demonstrating bioequivalence in pharmacokinetic parameters such as Cmax and AUC (Area Under the Curve), represent a cornerstone of sustainable pharmaceutical policy [1]. Regulatory bodies including the European Medicines Agency (EMA); the U.S. Food and Drug Administration (FDA),; and Poland’s Office for Registration of Medicinal Products, Medical Devices and Biocidal Products (URPL) have established rigorous standards to guarantee that generics are equivalent to originator in terms of safety, efficacy, and quality [2]. Despite this scientific consensus, the practical implementation of generic substitution remains inconsistent, shaped not only by regulatory frameworks but also by the perceptions and attitudes of healthcare professionals and patients.

Pharmacists are highly trusted providers whose acceptance of generics influences patient adherence, especially where substitution at the point of dispensing is permitted [3]. In Poland, pharmacists may offer a lower-cost reimbursed equivalent unless the prescriber prohibits substitution [4]. In practice, decisions reflect patient expectations and experiences as well as pharmacists’ beliefs and sociodemographic profile.

Attitudes toward generics are shaped by system and individual-level factors. Education on bioequivalence and regulatory approval tends to increase confidence, whereas limited training and concerns about narrow-therapeutic-index medicines or complex regimens foster caution; policy incentives may also conflict with professional judgment [5]. For some medicines, reviews note risks during switching (bioequivalence-related variability, loss of seizure control, adverse events), and guidelines advise that well-controlled patients avoid switching brand-to-generic, generic-to-brand, or between generics [6]. In Poland, detailed evidence remains scarce and the influence of sociodemographic factors is insufficiently characterized [7]. Accordingly, this study tests whether these characteristics are associated with pharmacists’ attitudes toward generics and their self-reported substitution practices.

Given the central role of pharmacists in the implementation of pharmaceutical policy, investigating their perceptions of generic versus originator offers valuable insight into how regulations are translated into everyday practice. A more nuanced understanding of the determinants of these perceptions can inform targeted educational programs, improve patient counseling practices, and support the refinement of reimbursement and substitution policies.

To address these gaps, we conducted a cross-sectional study among Polish pharmacists. The study aimed to assess their knowledge and attitudes toward the efficacy, safety, quality, and therapeutic equivalence of generic medicines, and to describe their behavioral intentions and self-reported substitution practices. In addition, we examined whether selected sociodemographic characteristics were associated with these outcomes.

## 2. Methods

### 2.1. Study Design and Setting

We conducted a cross-sectional, anonymous survey in collaboration with the Union of Pharmacy Employees (Związek Zawodowy Pracowników Farmacji, ZZPF) between 10 June and 31 August 2025.

### 2.2. Sampling Frame and Recruitment

To maximize reach and response rates, we used a multi-channel strategy. The questionnaire was disseminated during national and regional pharmaceutical conferences, training sessions, and educational meetings (direct electronic access on site; total 1200 participants). In parallel, a targeted email campaign was sent to pharmacists affiliated with or cooperating with ZZPF (about 3300 members). At selected events, trained staff with tablets facilitated on-site completion. QR codes linking to the survey were displayed prominently at venues to enable mobile participation. In total, 342 pharmacists completed the survey, corresponding to a response rate of 10.08% from conference recruitment and 6.7% from electronic invitations.

### 2.3. Eligibility Criteria

Inclusion criteria were: licensed pharmacists or pharmacy trainees/interns working in Poland, aged ≥18 years, able to complete the questionnaire in Polish, and providing electronic informed consent. Exclusion criteria were: duplicate entries (screened by timestamp/IP pattern and completion time), incomplete submissions lacking primary outcomes, and respondents not currently practicing in pharmacy settings.

### 2.4. Questionnaire Development and Validation

The instrument was developed from prior literature and expert input. Content validity was assessed by subject-matter experts; face validity and comprehensibility were evaluated in a pilot. Feedback from the pilot was incorporated by refining item wording, clarifying instructions, and removing ambiguities. The final questionnaire covered demographics, practice characteristics, knowledge and attitudes, and self-reported behaviors.

The questionnaire consisted of two parts. The general section (6 items) collected demographics and professional background (gender, age, years of experience, primary practice location by locality size, specialization). The pharmacist-specific section included:

− Reasons for recommending generics in long-term therapy and single-use prescriptions (multi-response items);
− Doubts about generic effectiveness (3-option item);
− Views on whether bioequivalence testing should be performed between generics (3-option item);
− Patient-reported lack of improvement and/or adverse events with generic and originator (multi-response items);
− Seven Likert statements (5-point, from “strongly disagree” to “strongly agree”) on competition/innovation, perceived equivalence in effectiveness and safety, patient interest in substitution, payer savings, and access to therapy.

Content validity was established by an expert panel (clinical pharmacology, pharmaceutical care, health policy) who reviewed items for clarity, relevance, and alignment with study aims. Face validity and feasibility were assessed in a pilot with four pharmacists; no substantive changes were required.

### 2.5. Ethical Aspects

This study was approved by the Bioethics Committee of the Poznan University of Medical Sciences (Resolution No. 492/25 of 4 June 2025). Participation was voluntary and fully anonymous; electronic informed consent was obtained prior to survey initiation.

### 2.6. Statistical Analysis

Statistical analyses were performed using the StatSoft. Inc. (Tulsa, OK, USA, 2017) STATISTICA software, version 13.0. Analyses followed a complete-case approach (no imputation). We note that non-random missingness could bias estimates and interpret affected results with caution. Quantitative variables were described using means, standard deviations, medians (interquartile ranges), and 95% confidence intervals. Categorical variables were presented as frequencies and percentages. The normality of distribution for quantitative variables was assessed using the Shapiro–Wilk test. Relationships between variables were examined using Pearson’s chi-square test, Cramér’s V, and the Mann–Whitney U test. A significance level of *p*  =  0.05 was adopted for all tests.

## 3. Results

Among the respondents, a significant majority were female (67.5%), while 31.0% identified as male. The age distribution was relatively balanced, with the highest representation in the 31–40 years age group (30.7%), followed by pharmacists aged 41–50 (24.1%), and 21–30 (17.6%). This suggests a presence of both mid-career and early-career professionals. Regarding work experience, 32.2% of pharmacists reported more than 20 years in the profession, 26.3% between 11–20 years, and 20.1% between 6–10 years. Only 13.3% had less than five years of professional experience. The respondents were employed in both urban and rural areas, with the majority working in large cities or towns with over 50,000 inhabitants (Table 1).

Among the 342 pharmacists participating in the study, the majority (85.0%, n = 290) did not hold a specialization, while 15.0% (n = 51) declared having completed a postgraduate specialization in pharmacy. Among those with a specialization, the most frequently reported field was general pharmacy, accounting for 76.5% (n = 39) of the specialized subgroup. Other specializations included clinical pharmacy (15.7%, n = 8), hospital pharmacy (13.7%, n = 7), industrial pharmacy (2.0%, n = 1), pharmaceutical analytics (2.0%, n = 1), bromatology (2.0%, n = 1), and public health (2.0%, n = 1). Some pharmacists held more than one specialization, which may slightly affect the totals presented.

Regarding the factors influencing the recommendation of generic medicines in long-term therapies, pharmacists most frequently indicated financial considerations (91.0%). Safety of use (51.5%) and therapeutic efficacy (53.0%) were also commonly cited. Approximately 6% admitted they never recommend generics in chronic treatments (Table 2). Importantly, in the context of long-term therapy, several differences were observed in the use of generic medicines. Cost considerations were reported more frequently by respondents working in community pharmacies (χ^2^(1) = 4.14, *p* = 0.042). Effectiveness, by contrast, was more often indicated by respondents employed in hospital pharmacies (χ^2^(1) = 4.26, *p* = 0.039). Individuals who do not propose generic medicines were older (Mann–Whitney U test: U = 1250.00, *p* = 0.006) and had longer professional tenure (Mann–Whitney U test: U = 1332.50, *p* = 0.012).

When asked about their decision-making in the case of one-time prescriptions (e.g., antibiotics or painkillers), pharmacists also pointed to cost (76.2%) as a key determinant. However, the percentage of pharmacists emphasizing effectiveness (47.5%) and safety (45.0%) was slightly lower than in chronic conditions. Notably, 4.6% reported that they never offer generics in one-time prescriptions (Table 3). Cost reduction was more frequently indicated by women (χ^2^(2) = 2.70, *p* = 0.259), while effectiveness was more often cited by hospital pharmacy staff (χ^2^(1) = 3.45, *p* = 0.063) and by individuals with longer professional tenure (Mann–Whitney U test: U = 8534.50, *p* = 0.043). Individuals who do not propose generic medicines were older (Mann–Whitney U test: U = 940.00, *p* = 0.015) and had longer work experience (Mann–Whitney U test: U = 1122.00, *p* = 0.029).

Based on the responses from pharmacists regarding the effectiveness of generic medicines, over half of the participants (52.3%) reported never having doubts about the effectiveness of a generic medicine compared to its original counterpart. However, 36.2% admitted that they had experienced such doubts, while 11.5% had no clear opinion on the matter. These doubts were more frequently reported by women (χ^2^(4) = 10.17, *p* = 0.038), by staff working in hospital pharmacies (χ^2^(2) = 16.40, *p* < 0.001), and by respondents with a specialty qualification (χ^2^(2) = 15.57, *p* < 0.001). Among those who indicated they had doubts about the effectiveness of generics, 53.5% stated that, in such cases, they would recommend switching to a different medicine, even if it were more expensive. In contrast, 27.7% would not suggest a switch, and 18.8% had no opinion on the issue.

In the standard procedure for introducing a generic medicine, bioequivalence is assessed against the reference (innovator) product. We asked whether bioequivalence studies should also be conducted between generics containing the same active substance, rather than only versus the originator. Among 280 respondents, nearly half supported this approach (49.6%), 29.6% were opposed, and 20.7% had no opinion, indicating clear support but not full consensus.

The perceptions and feedback received from patients also played a role in pharmacists’ attitudes. When asked whether patients had ever reported that generic drugs were less effective, 58.5% of pharmacists confirmed such occurrences. Additionally, 73.4% had received reports of side effects after switching to generics, while 12.1% noted that patients had explicitly stated a lack of adverse reactions. These findings suggest that patient experiences, whether grounded in clinical effects or psychological expectations, may influence pharmacists’ willingness to suggest generics.

The majority of respondents believe that the presence of generic drugs supports innovation in the industry: a total of 61.4% (17.1% strongly agree, 44.3% rather agree). A total of 19.3% have no opinion and 19.2% disagree (including 4.6% strongly disagree).

Most respondents agreed that generics are as effective as originators (82.9%: 63.6% “rather agree,” 19.3% “strongly agree”); 10.4% disagreed and 6.8% had no opinion. Perceived safety was slightly higher (84.6%: 60.0% “rather agree,” 24.6% “strongly agree”), with 8.5% disagreeing and 6.8% undecided (Table 4). Both the efficacy and safety of generic drugs were better assessed by pharmacists working in community pharmacies (χ^2^(4) = 12.59, *p* = 0.013; χ^2^(4) = 24.98, *p* < 0.001, respectively).

The vast majority of respondents believe that the emergence of generics allows for savings on reimbursement and allows for the expansion of the drug list: 73.6% agree, 18.2% have no opinion, and 8.2% disagree. An even greater consensus concerns easier patient access to pharmacotherapy (including through lower prices and greater availability of molecules): 92.5% agree, 3.6% have no opinion, and 3.9% disagree (Table 5).

A statistically significant association was observed only between agreement that generic medicines increase patients’ access to pharmacotherapy and workplace setting. Pharmacists working in community pharmacies were significantly more likely to agree with this statement (χ^2^(4) = 15.58, *p* = 0.004).

## 4. Discussion

This study provides a cross-sectional analysis of Polish pharmacists’ attitudes toward branded and generic medicines, as well as the role of sociodemographic factors in shaping these opinions and substitution practices. The overall picture is positive: the vast majority of respondents recognized the therapeutic equivalence and safety of generics (82.9% and 84.6% agreement, respectively), and these assessments were significantly more favorable in community pharmacies (efficacy: *p* = 0.013; safety: *p* < 0.001). These results are consistent with previous reports emphasizing the key role of pharmacists in building acceptance of generics among prescribers and patients [8].

At the same time, areas of hesitation emerged. In long-term therapy, the most frequently cited reason for recommending generics was cost (91.0%), with cost emphasis appearing more frequently in community pharmacies (*p* = 0.042), while efficacy was more frequently emphasized by hospital pharmacists (*p* = 0.039). Across the entire sample, 36.2% of respondents declared they had experienced doubts about the effectiveness of generics; more than half of them (53.5%) would recommend switching therapy, even to a more expensive one, in such a situation. In practice, pharmacists also frequently received reports from patients about lower efficacy (58.5%) and adverse events following the switch (73.4%). This type of caution, especially with drugs with a narrow therapeutic index is consistent with previous observations of the risk of treatment destabilization and worsening health outcomes in selected therapeutic groups [9,10].

Age was one of the most important differentiating factors: those who did not recommend generics were significantly older and had longer experience (*p* = 0.006; *p* = 0.012). In single-use prescriptions, longer experience was also associated with a more frequent emphasis on efficacy (*p* = 0.043). This pattern corresponds to generational differences in exposure to content about bioequivalence, regulations, and pharmacoeconomics [11]. Gender differences were less pronounced: women were more likely to cite cost as a factor in decisions regarding single-use medications, although without statistical significance (*p* = 0.259), while they were more likely to report concerns about efficacy (*p* = 0.038). Converging reports link greater sensitivity to cost barriers and adherence with a patient-centric orientation among female professionals [12,13,14].

Workplace context also influenced attitudes and practices. In our data, pharmacists from community pharmacies were more likely to emphasize costs and agree that generics improve access to medication (92.5% agreement across the entire sample; association with workplace *p* = 0.004), whereas hospital pharmacists were more likely to emphasize efficacy. Consistent with the literature, the hospital environment promotes a greater focus on protocols and evidence, while the community pharmacy environment exposes patients to resistance and concerns at the counter [15].

It is worth emphasizing the gap between knowledge and practice: despite a high level of agreement on equivalence, some pharmacists, faced with patient preferences give up with substitution to maintain trust and continuity of therapy [16]. Similar tensions between systemic goals and patient expectations have been noted in Australia and the United Kingdom [7,17,18]. Furthermore, although support for INN (International Nonproprietary Name) prescribing is sometimes expressed, respondents point to implementation barriers on the part of prescribers and a lack of enforcement mechanisms, which limits the effectiveness of policies promoting INN [4].

Education and professional development strengthen confidence in generics: additional training in the areas of regulation, pharmacoeconomics, and drug policy is associated with greater support for substitution and better communication with patients [19]. This calls for the systematic inclusion of content about generics in continuing education and training programs, as well as interprofessional activities connecting pharmacists and physicians [20]. At the same time, respondents noted the lack of standardized, accessible materials explaining bioequivalence and ambiguous labeling, which complicates working with older individuals and those with lower health literacy [21]. The phenomenon of misinformation, reinforced by anecdotes, online forums, and media coverage, further complicates dialogue in pharmacies, indicating the need for coordinated public health campaigns [22].

From a broader perspective, respondents despite recognizing the economic importance of generics (including 73.6% agreement that they generate savings for payers and enable the expansion of lists) highlighted the vulnerability of supply chains and the need for policies ensuring supply continuity and transparent purchasing mechanisms, which were exacerbated by recent global disruptions [23,24]. Overall, generic adoption remains a multifactorial phenomenon, dependent on individual competencies, patient trust, institutional support, and system resilience [25]. Effective actions should combine targeted education (with particular emphasis on hospital communities and specialists), policy reforms (including effective INN support), patient communication standards, and strengthening supply chains [26,27].

### Limitations of the Study

First, the cross-sectional design and reliance on self-reported data may introduce self-report (including recall and social desirability) bias and preclude causal inference. Second, selection bias cannot be ruled out: recruitment through conferences, trainings, and ZZPF channels, together with the over-representation of community pharmacists and women, may limit generalizability to the broader pharmacist workforce. Third, as this is an exploratory study, we did not adjust for multiple comparisons; thus some associations, especially weaker one, may reflect Type I error rather than true relationships and should be interpreted with caution.

## 5. Conclusions

The study indicates that Polish pharmacists’ attitudes toward generic drugs are generally positive: most recognize their therapeutic equivalence and safety compared to brand-name drugs, and lower costs remain the primary reason for recommending generics for long-term therapy. The context of this study differentiates assessments and decisions: in community pharmacies, cost was more frequently emphasized, and generics’ efficacy and safety were rated higher, while in hospital pharmacies, efficacy was more often emphasized as a selection criterion. Personal and professional characteristics also play a role, those who did not recommend generics were older and had more experience; doubts about efficacy were more frequently expressed by women, hospital pharmacists, and specialists. At the same time, respondents broadly recognize the systemic benefits (payer savings, improved access to pharmacotherapy), and nearly half support conducting bioequivalence studies, including between generics.

In clinical practice, this implies the need for targeted actions: strengthening education, standardizing patient communication materials, and simplifying labeling, as well as concurrently strengthening INN prescriptions and direct patient communication tools. Given the limitations of our study, further work should combine self-reports with objective measures of behavior and clinical outcomes and evaluate the effectiveness of educational and communication interventions in various practice settings.

## Figures and Tables

**Table 1:** Basic characteristics of the study group in terms of gender, age, work experience and place of work.

	Pharmacists (n = 342)
Gender	
man	106 (31.0%)
woman	231 (67.5%)
I prefer not to answer	5 (1.5%)
Age [years]	
average (SD)	39.1 (9.8)
scope	25.0–70.0
median (IQR)	38.0 (14.5)
95%CI	[38.0; 40.1]
Practice [years]	
average (SD)	13.1 (9.1)
scope	0.0–42.0
median (IQR)	12.0 (15.0)
95%CI	[12.1; 14.1]
Place of work *	
Community pharmacy	256 (74.9%)
Hospital pharmacy	75 (21.9%)
Other	20 (5.8%)
Place of professional activity	
City over 500 thousand inhabitants	138 (40.2%)
City 100–500 thousand inhabitants	76 (22.3%)
City 50–100 thousand inhabitants	39 (11.4%)
City 10–50 thousand inhabitants	56 (16.4%)
City up to 10 thousand inhabitants	19 (5.6%)
Countryside	14 (4.1%)

* Multiple answer option.

**Table 2:** Considerations taken into account when proposing a generic medicine as the initial treatment option for long-term pharmacotherapy (n = 266).

	Category	Cardinality	Percent (%)
Reducing treatment costs	Yes	242	91.0
	No	24	9.0
Safety of pharmacotherapy	Yes	137	51.5
	No	129	48.5
The effectiveness of pharmacotherapy	Yes	141	53.0
	No	125	47.0
Other option (please describe)	Yes	23	8.6
	No	243	91.4
I do not recommend generic drugs	Yes	16	5.7
	No	266	94.3

**Table 3:** Considerations taken into account when proposing a generic medicine for single-use treatment (n = 282).

	Category	Cardinality	Percent (%)
Reducing treatment costs	Yes	215	76.2
	No	67	23.8
Safety of pharmacotherapy	Yes	127	45.0
	No	155	55.0
The effectiveness of pharmacotherapy	Yes	134	47.5
	No	148	52.5
Other option (please describe)	Yes	35	12,4
	No	247	87.6
I do not recommend generic drugs	Yes	13	4.6
	No	269	95.4

**Table 4:** Perceptions of Generics’ Effectiveness and Safety (% of respondents).

	Effectiveness: “Generic Drugs Are as Effective as Original”	Safety: “Generic Drugs Are as Safe as Original”
Strongly agree	19.3%	24.6%
Rather agree	63.6%	60.0%
No opinion	6.8%	6.8%
Rather disagree	8.6%	6.4%
Strongly disagree	1.8%	2.1%

**Table 5:** Opinions on the impact of generic drugs on system savings and patient access to pharmacotherapy (% of respondents).

	Savings and Expanding Reimbursement	Access to Pharmacotherapy
Strongly agree	37.9%	51.8%
Rather agree	35.7%	40.7%
No opinion	18.2%	3.6%
Rather disagree	5.7%	1.8%
Strongly disagree	2.5%	2.1%

## Data Availability

The data presented in this study are available on request from the corresponding author due to privacy/ethical concerns. Because responses are linked to professional registration details, a small residual risk of indirect identification remains even after de-identification. In addition, participants consented to use by the study team only, and the organizer’s agreement does not cover public data sharing.
